# Advances in the Isolation of Specific Monoclonal Rabbit Antibodies

**DOI:** 10.3389/fimmu.2017.00494

**Published:** 2017-05-05

**Authors:** Zaibao Zhang, Huijuan Liu, Qian Guan, Lei Wang, Hongyu Yuan

**Affiliations:** ^1^Institute for Conservation and Utilization of Agro-Bioresources in Dabie Mountains, Xinyang Normal University, Xinyang, China; ^2^College of Life Science, Xinyang Normal University, Xinyang, China; ^3^State Key Laboratory of Organ Failure Research, National Clinical Research Center for Kidney Disease, Division of Nephrology, Nanfang Hospital, Southern Medical University, Guangzhou, China

**Keywords:** rabbit antibody repertoire, rabbit monoclonal antibody, hybridoma, phage display, single B cell antibody technology

## Abstract

The rabbit monoclonal antibodies (mAbs) have advantages in pharmaceuticals and diagnostics with high affinity and specificity. During the past decade, many techniques have been developed for isolating rabbit mAbs, including single B cell antibody technologies. This review describes the basic characterization of rabbit antibody repertoire and summarizes methods of hybridoma technologies, phage display platform, and single B cell antibody technologies. With advances in antibody function and repertoire analysis, rabbit mAbs will be widely used in therapeutic applications in the coming years.

## Introduction

Monoclonal antibodies (mAbs) are essential tools in biochemistry, molecular biology, and medicine research. mAb therapeutics has revolutionized the approach to many serious human diseases with an increasing speed, and over 230 mAbs were evaluated in phase clinical studies in early 2017 (1). Many methods exist for the generation and identification of mAbs for both research and therapeutic purposes. The mouse hybridoma method described by Kohler and Milstein in 1975 was the first and most widely used approach for obtaining mouse mAbs (2). In the past few decades, several display techniques such as phage display (3), yeast surface display, ribosome display (4, 5), and mRNA display technologies (6) have been used for producing mAbs. Although these antibody generation technologies were widely adopted for mAbs screening, these methods were inefficient and required time-consuming operations. In addition, the natural cognate pairing information of antibodies is lost in display methods, which reduced the specific diversity of antibodies.

More recently, a single B cell-based method has been developed that allows direct sampling of the immune repertoire from a single B-cell or the clonally expanded progeny of single cell (7). This technology avoids the inefficient hybridoma fusion step and retains the natural heavy and light chain pairing, thereby allowing a more thorough interrogation of the B cell population. Moreover, this technique exploits the natural process of affinity, specificity, and stability profiles of mAbs. Antigen-specific memory B cells expressing surface IgG and IgG-secreting plasma cells have been exploited extensively as a source of mAbs. The methods for single-cell isolation are currently reliant on fluorescence-activated cell sorting (FACS) and manual micromanipulation, and the antibody genes were transferred to mammalian cells for mAb expression and further characterization (8, 9).

The rabbit immune system generates antibody diversity and optimizes affinity by mechanisms different from those of mice and other rodents (10). Compared with traditional mouse mAbs, the rabbit mAbs have advantages in diagnostics with high affinity and specificity toward antigens, more diverse epitope recognition, and greatly improved response to small-size epitopes and mouse antigens (11). Owing to these favorable features, more than 9,519 rabbit mAbs were generated by Abcam, which were identified for major signaling pathways including apoptosis, cell cycle, epidermal growth factor receptor signaling, and transforming growth factor-β signaling. Recently, some new platforms have been developed for generating rabbit mAbs from antigen-specific memory B cells or plasma cells by FACS or manual micromanipulation, and expression of recombinant mAbs in prokaryotic, eukaryotic, or cell-free expression systems (9, 12–15). These technology platforms have proven to be efficient and robust for generation of large panels of antigen-specific recombinant antibodies from immunized rabbits within 1 week, which promoted the production of rabbit mAbs.

## The Rabbit Antibody Repertoire

The rabbit immunoglobulin heavy chain (IGH) locus contains over 200 IGH variable germline genes, with over 50% have been found to be “non-functional” (16, 17). Also, more than 50 IG Kappa V and 17 IG Lambda V functional genes were identified with genomic sequencing (IMGT database). The diversity of rabbit VH repertoire is more limited, and the V light (VL) repertoire is more diverse than that in mice and humans (17–20). Most rabbit antibodies are derived from the IGHV1 gene (21). In addition, the rabbit VL repertoire displays a larger complementarity-determining region (CDR)-L3 loop length than its human and mouse counterparts (22).

Recently, the comprehensive rabbit antibody repertoire was analyzed with next-generation sequencing (NGS) technology (22). In this study, IGHV1S40 and IGHV1S45 were found to dominate the VH repertoire of naïve rabbit, while IGHV1S69 contributed significantly to immunized rabbit with a 16-mer peptide (22). In addition, the somatic mutations in VH and VL are higher than its human and mouse counterparts, with VH regions accumulating two-third more mutations than humans and mice and VL regions accumulating more mutations in fragment region (FR)-1 and FR-3. The average length of rabbit CDR-H3 is 12 amino acids, which is similar to its human and mouse counterparts, while the CDR-L3 length is much longer than its human and mouse counterparts. Rabbit immune system uses both gene conversion and somatic hypermutation to diversify antibody repertoire (18), and more mutations in rabbits repertoire compared with humans and mice could compensate for the limited diversity of the germline genes used to build the rabbit functional repertoire (22).

## Hybridoma Method

**Figure 1:**
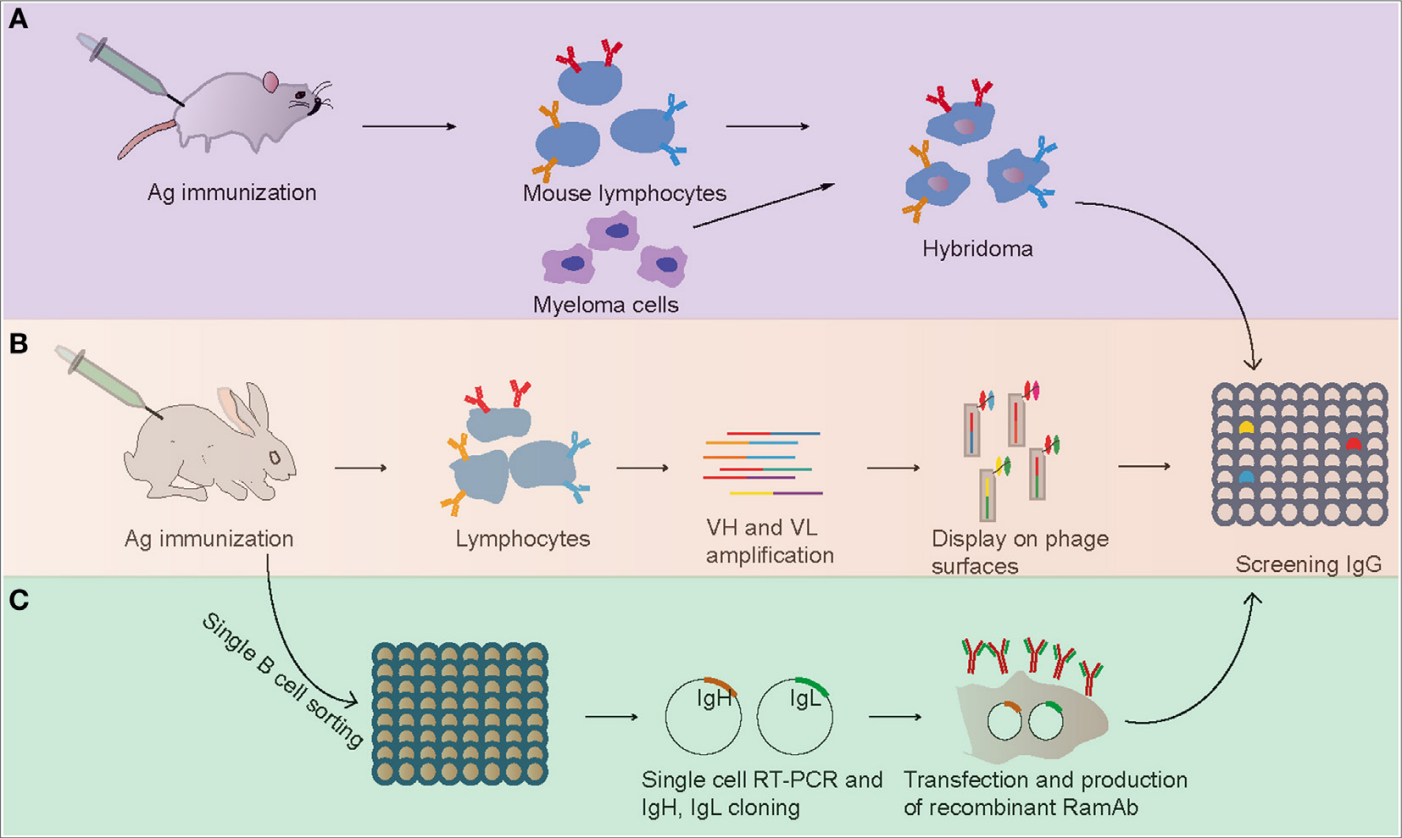
**Flowchart summarizing the generation of antigen-specific monoclonal antibodies**. **(A)** Mouse hybridoma method. **(B)** Phage display method. **(C)** Single B cell antibody technology.

Mouse hybridoma technology, which depends on cell fusion of B cell to myeloma partner, is a traditional and most widely and successfully used approach for the production of mouse mAbs since its identification in 1975 (Figure 1) (2). In this method, the lymphocytes from immunized mice were harvested and fused with myeloma cell derived from the BALB/c mouse to form an immortalized hybridoma cell (2). Then, the hybridoma cells were screened to identify specific clones producing identical antibodies (Figure 1). The rabbit mAbs were first reported in 1988 with mouse–rabbit heterohybridomas method (23). However, this mouse–rabbit heterophybridomas were relatively inefficient, unstable, and unable to secrete antibodies for prolonged periods (11). In the mid-1990s, the stable rabbit–rabbit hybridomas were generated to produce rabbit mAbs (24). These rabbit–rabbit hybridomas also proved to be less stable than conventional mouse hybridomas and that limited their widely use at laboratory level. Moreover, due to inefficient fusion (5 × 10^−6^ efficiency with conventional polyethylene glycol fusion) and transformation events, the application of hybridoma technologies for production of rabbit mAbs was limited.

Hybridoma technology is a well-established method to generate murine mAbs and is widely used to produce antibodies for a variety of applications. However, due to lack of a suitable myeloma partner, hybridoma method is mostly restricted to rodent immunizations. In the 1980s of the twentieth century, a human hybridoma technology for production of therapeutic antibodies that permits generation of natural human antibodies in a native form was developed (25). This human hybridoma technology developed another useful way of generating therapeutic antibodies without additional modifications. Numerous studies were devoted to this technology, and several useful human fusion partner cell lines were discovered (26). Based on the development of better fusion partners and technical advances in using electrofusion, the success rate of human hybridoma fusion was improved, which can prompt the development of therapeutic antibodies in the nearest future (26). Interestingly, a genetic modification method was developed recently to immortalization of human B cells by overexpressing BCL-6 and BCL-XL (27). By using this technology, the group of Spits has isolated a number of mAbs against a variety of pathogenic viruses (27). In addition, this technology was successfully applied in other species, and rabbit mAbs were efficiently generated from immortalized memory B cells. Therefore, this technology developed a new interesting and convenient antibody discovery platform, which will become the mainstream method for mAbs production.

## Phage Display

From the early 1990s, phage display technology was explored as a new method for generating mAbs (28, 29). In this approach, the V gene repertoires were harvested from lymphocytes, and the combinations of VHs and VLs were cloned and expressed on the surface of filamentous bacteriophage by fusion to its coat protein (Figure 1). Then, phage particles bearing expressed specific mAbs on their tips were selected and used in conventional assays. Compared with the hybridoma technology, which was practically confined to rodents, phage display has been successfully used to screen and isolate mAbs from any species whose immunoglobulin genes are known (30, 31). In 2000, Rader et al. first described the whole process of selecting rabbit mAbs with phage display technology (32). This technology was successfully used in selecting and humanizing rabbit antibodies against human A33 antigen, a target antigen for the immunotherapy of colon cancer, and the obtained humanized antibodies retained high specificity and affinity for human A33 antigen (32). At present, antibody phage display is a major technical platform to generate fully human antibodies for therapeutic purpose for its high speed and ease of antibody generation and the ability to control various selection parameters *in vitro* (33).

Dependent on our knowledge of antibody structure, function, and sequence diversity, in addition to the insights from previous antibody libraries, a new synthetic antibody library technology was developed and used in mAb production by inserting precisely designed sequences into the antigen-combining site (34, 35). This technology is accomplished based on genetically copying IgG fragments and on using molecular biology techniques to diversify antibody paratopes *in vitro* (36). Several synthetic antibody libraries (e.g., HuCAL and Ylanthia library) with multiple variable heavy and light chain framework regions were designed and used to isolate human mAbs optimized for molecular recognition (37–40). With advances in library designs and selection methods, synthetic antibody libraries will become an indispensable tool for rapid generation of target-binding mAbs with exquisite specificity.

## Generation of mAbs from Single Rabbit B Cells by Single-Cell Cloning

Although hybridoma screening and display methodologies have been used in rabbit mAbs production, they both have some drawbacks: the hybridoma technology has a low efficiency of cell fusion, while display method results in the loss of natural cognate pairing of heavy and light chain (7). To overcome these issues, a single B cell antibody technology has been developed recently (Figure 1) (7). Briefly, single B cell antibody technology consists of the following short steps: (i) identification and isolation of specific single B cells with random way or FACS from peripheral blood or from lymphoid tissues, (ii) single-cell reverse transcription-polymerase chain reaction (RT-PCR) with antibody-specific primers, (iii) amplification of Ig genes with PCR and sequencing, (iv) cloned the Ig genes into expression vector, (v) Ig genes were expressed in bacterial systems (e.g., *Escherichia coli*) or mammalian cell systems (e.g., HEK 293, CHO cells), and (vi) proteins were purified and evaluated with enzyme-linked immunosorbent assay (Figure 1). This method has been widely used in human and mouse mAb production and yielded some therapeutic neutralizing mAbs for several diseases including cancer, autoimmune disorders, and infectious diseases (7). For example, several valuable mAbs were isolated from HIV patients with single B cell antibody technology, which will be useful for clinical diagnostics (41). The most important advantage of this approach is preserving the natural cognate pairing of heavy and light chain, allowing exploitation of the natural process of antibody affinity, specificity, and maturation. Therefore, this technology favors the generation of mAbs with an attractive affinity, specificity, and stability profile.

Memory B cells and plasma/plasmablast cells are main sources of antibody production. Most methods adopted in single B cell antibody analysis in human, mice, and rabbits were focused on memory B cell or plasma/plasmablast cells. Kurosawa et al. developed an endoplasmic reticulum (ER)-based method for identification and isolation of antigen-specific plasma/plasmablast cells with FACS and ER-specific fluorescent dye (12). This method enhanced the selection efficiency of plasma/plasmablast and eliminated cell propagation and screening processes. In addition, Ozawa et al. developed an immunospot array assay on a chip technology to detect rabbit antigen-specific mAbs, and this technology could produce rabbit mAbs that specifically recognize a phosphorylation site-specific epitope of human transforming growth factor-β-activated kinase 1 (42). Clargo et al. reported a fluorescence-based method combination with micromanipulation to isolate rabbit single antigen-specific IgG-secreting plasma cells for subsequent antibody expression analysis (9). Also, Seeber et al. used a lymphocyte panning to capture the antigen-specific B cells in combination with *in vitro* B cell short cultivation and B cell cloning to isolate functional rabbit mAbs from peripheral blood (13).

The isolation of rabbit lymphocytes was limited by lack of useful surface markers. To select the rare and specific rabbit B cells for mAb expression, several new methods were developed. Recently, Starkie et al. described a two-color antigen staining method to identify antigen-specific rabbit memory B cells with an efficient antibody identification of 38.5% (43). In this work, negative cell staining was used to eliminate T cells, naïve IgM^+^ B cells, and dead cells, and positive cell staining with anti-IgG and a dual-antigen labeling step (FITC and PE) allowed identification of the antigen-specific class-switched IgG^+^ memory B cell subset (43). This FACS technology incorporation with antigen-binding step enhanced the recovery of antigen-specific mAbs and was therefore high throughput. In addition, another simple and flexible method, HybriFree, was developed and successfully used in mouse, rabbit, and chicken mAbs production (14). The HybriFree workflow includes four steps: capturing antigen-specific B cell with antigen-loaded solid matrix, single-cell amplification of VH and VL coding cDNAs, construction of a combinatorial VH–VL library in mammalian expression system, and determination of proper VH–VL combinations (14). This method was successfully used in the production of rabbit mAbs against mouse CD48 with high efficiency within 10 days and can apply to any species whose antibody cDNA sequence is available (14). Another novel screening platform called “single-cell RT-PCR linked *in vitro* expression (SICREX)” for rabbit mAb production was described (15). In this work, antigen-specific B cells were isolated with micromanipulation combined with antigen-conjugated magnetic beads, and Ig genes were amplified for cell-free production with a linear Ig expression cassette. The linear expression cassette contains all essential elements for transcriptional and translational regulation, including T7 promoter and T7 terminator, and fragment of antigen-binding or single-chain variable fragment was expressed without cloning. Compared with the previously platform, the entire process of SICREX is conducted *in vitro*, which reduced the mAb production time to a few days.

Recently, mass spectrometry (MS)-based *de novo* sequencing approaches have been used for the identification of purified mAbs from serum-derived polyclonal antibody pool, which prompted the development of proteomic deconvolution of serum Igs (44). Combined with advancements in NGS and MS technologies, this method was successfully used in determination of the antibody composition of the polyclonal serum response after immunization in rabbit (45). In this approach, full-length serum IgGs were purified and treated with pepsin to prepare F(ab)_2_ fragments, and then antigen-specific F(ab)_2_ fragments were isolated and proteolytically digested for liquid chromatography high-resolution tandem-MS (LC-MS/MS). In parallel, an antibody repertoire database was constructed and sequenced by NGS. By mapping peptides with antibody database, the individual mAbs were identified (45).

## Future Trends

Monoclonal antibodies are widely used in the fields of therapeutic, diagnostic, and biological applications due to their high specificity and affinity. At present, the majority of mAbs to human antigens were generated in mice, and mouse monoclonal antibodies (MMAs) have been exploited widely in diagnostic and therapeutic settings (1, 12). However, many disadvantages of using MMAs as immunotherapeutic agents for humans, including short half-life time in serum and induction of human anti-mouse antibodies reaction, have been reported (46, 47). In addition, it is well known that many human immunogens failed to stimulate antibody responses in mice. Theses disadvantages limit the use of MMAs in therapeutic and diagnostic application. Compared to conventional MMAs, rabbit mAbs are more ideal for use in research and diagnosis. Rabbit mAbs exhibit several combined benefits including high affinity and specificity toward antigens, more diverse epitope recognition, and greatly improved response to small-size epitopes and mouse antigens (11, 48, 49). In addition, rabbits are evolutionarily distant from mice, which can produce antibodies to some antigens that are not immunogenic in mice (50, 51). These advantages made rabbit mAbs attractive diagnostic reagents in mouse models of human diseases. Similar to mouse mAbs, the rabbit mAbs were also displayed several limitations including inducing immunogenicity in humans. In an attempt to reduce the immunogenicity and to prompt the use of rabbit mAbs for therapeutic applications, antibody engineering methods such as chimerization, humanization, and Fc engineering were successfully applied. Two previous researches had showed that the humanized rabbit mAbs retained the high specificity and affinity to human antigen, which can be used in diagnostic applications (10, 32). Also, a comparative study between rabbit mAbs and the corresponding MMAs on several tumor types showed that rabbit mAbs displayed an increased sensitivity with no apparent loss of specificity (11). Therefore, the rabbit immune system is an important source for the generation of therapeutic human antibodies to human antigens, and the humanized rabbit mAbs will be widely used in therapeutic applications in the future.

## Author Contributions

ZZ wrote the manuscript. ZZ, HL, QG, LW, and HY approved its final version.

## Conflict of Interest Statement

The authors declare that the research was conducted in the absence of any commercial or financial relationships that could be construed as a potential conflict of interest.

## References

[1] Reichert JM. Antibodies to watch in 2017. MAbs (2017) 9:167–81. 10.1080/19420862.2016.1269580 27960628 PMC5297518

[2] Kohler G Milstein C Continuous cultures of fused cells secreting antibody of predefined specificity. Nature (1975) 256:495–7. 10.1038/256495a0 1172191

[3] Smith GP. Filamentous fusion phage: novel expression vectors that display cloned antigens on the virion surface. Science (1985) 228:1315–7. 10.1126/science.4001944 4001944

[4] Murai T Ueda M Yamamura M Atomi H Shibasaki Y Kamasawa N Construction of a starch-utilizing yeast by cell surface engineering. Appl Environ Microbiol (1997) 63:1362–6. 9097432 10.1128/aem.63.4.1362-1366.1997 PMC168429

[5] Rakestraw JA Aird D Aha PM Baynes BM Lipovsek D. Secretion-and-capture cell-surface display for selection of target-binding proteins. Protein Eng Des Sel (2011) 24:525–30. 10.1093/protein/gzr008 21402751

[6] Roberts RW Szostak JW. RNA-peptide fusions for the in vitro selection of peptides and proteins. Proc Natl Acad Sci U S A (1997) 94:12297–302. 10.1073/pnas.94.23.12297 9356443 PMC24913

[7] Tiller T Single B cell antibody technologies. N Biotechnol (2011) 28:453–7. 10.1016/j.nbt.2011.03.014 21473940 PMC7102800

[8] Smith K Garman L Wrammert J Zheng NY Capra JD Ahmed R Rapid generation of fully human monoclonal antibodies specific to a vaccinating antigen. Nat Protoc (2009) 4:372–84. 10.1038/nprot.2009.3 19247287 PMC2750034

[9] Clargo AM Hudson AR Ndlovu W Wootton RJ Cremin LA O’Dowd VL The rapid generation of recombinant functional monoclonal antibodies from individual, antigen-specific bone marrow-derived plasma cells isolated using a novel fluorescence-based method. MAbs (2014) 6:143–59. 10.4161/mabs.27044 24423622 PMC3929438

[10] Steinberger P Sutton JK Rader C Elia M Barbas CF III. Generation and characterization of a recombinant human CCR5-specific antibody. A phage display approach for rabbit antibody humanization. J Biol Chem (2000) 275:36073–8. 10.1074/jbc.M002765200 10969070

[11] Rossi S Laurino L Furlanetto A Chinellato S Orvieto E Canal F Rabbit monoclonal antibodies: a comparative study between a novel category of immunoreagents and the corresponding mouse monoclonal antibodies. Am J Clin Pathol (2005) 124:295–302. 10.1309/NR8HN08GDPVEMU08 16040303

[12] Kurosawa N Yoshioka M Fujimoto R Yamagishi F Isobe M. Rapid production of antigen-specific monoclonal antibodies from a variety of animals. BMC Biol (2012) 10:80. 10.1186/1741-7007-10-80 23017270 PMC3520816

[13] Seeber S Ros F Thorey I Tiefenthaler G Kaluza K Lifke V A robust high throughput platform to generate functional recombinant monoclonal antibodies using rabbit B cells from peripheral blood. PLoS One (2014) 9:e86184. 10.1371/journal.pone.0086184 24503933 PMC3913575

[14] Kivi G Teesalu K Parik J Kontkar E Ustav M Jr Noodla L HybriFree: a robust and rapid method for the development of monoclonal antibodies from different host species. BMC Biotechnol (2016) 16:2. 10.1186/s12896-016-0232-6 26747451 PMC4706699

[15] Ojima-Kato T Hashimura D Kojima T Minabe S Nakano H. In vitro generation of rabbit anti-*Listeria monocytogenes* monoclonal antibody using single cell based RT-PCR linked cell-free expression systems. J Immunol Methods (2015) 427:58–65. 10.1016/j.jim.2015.10.001 26454028

[16] Currier SJ Gallarda JL Knight KL. Partial molecular genetic map of the rabbit VH chromosomal region. J Immunol (1988) 140:1651–9. 3126232

[17] Ros F Puels J Reichenberger N van Schooten W Buelow R Platzer J. Sequence analysis of 0.5 Mb of the rabbit germline immunoglobulin heavy chain locus. Gene (2004) 330:49–59. 10.1016/j.gene.2003.12.037 15087123

[18] Mage RG Lanning D Knight KL B cell and antibody repertoire development in rabbits: the requirement of gut-associated lymphoid tissues. Dev Comp Immunol (2006) 30:137–53. 10.1016/j.dci.2005.06.017 16098588

[19] Sehgal D Johnson G Wu TT Mage RG. Generation of the primary antibody repertoire in rabbits: expression of a diverse set of Igk-V genes may compensate for limited combinatorial diversity at the heavy chain locus. Immunogenetics (1999) 50:31–42. 10.1007/s002510050683 10541804

[20] Ros F Reichenberger N Dragicevic T van Schooten WC Buelow R Platzer J. Sequence analysis of 0.4 megabases of the rabbit germline immunoglobulin kappa1 light chain locus. Anim Genet (2005) 36:51–7. 10.1111/j.1365-2052.2004.01221.x 15670131

[21] Knight KL. Restricted VH gene usage and generation of antibody diversity in rabbit. Annu Rev Immunol (1992) 10:593–616. 10.1146/annurev.immunol.10.1.593 1590997

[22] Kodangattil S Huard C Ross C Li J Gao H Mascioni A The functional repertoire of rabbit antibodies and antibody discovery via next-generation sequencing. MAbs (2014) 6:628–36. 10.4161/mabs.28059 24481222 PMC4011907

[23] Raybould TJ Takahashi M. Production of stable rabbit-mouse hybridomas that secrete rabbit mAb of defined specificity. Science (1988) 240:1788–90. 10.1126/science.3289119 3289119

[24] Spieker-Polet H Sethupathi P Yam PC Knight KL. Rabbit monoclonal antibodies: generating a fusion partner to produce rabbit-rabbit hybridomas. Proc Natl Acad Sci U S A (1995) 92:9348–52. 10.1073/pnas.92.20.9348 7568130 PMC40982

[25] Olsson L Kaplan HS. Human-human hybridomas producing monoclonal antibodies of predefined antigenic specificity. Proc Natl Acad Sci U S A (1980) 77:5429–31. 10.1073/pnas.77.9.5429 6159646 PMC350072

[26] Glukhova XA Prusakova OV Trizna JA Zaripov MM Afanas’eva GV Glukhov AS Updates on the production of therapeutic antibodies using human hybridoma technique. Curr Pharm Des (2016) 22:870–8. 10.2174/1381612822666151223102845 26696411

[27] Kwakkenbos MJ van Helden PM Beaumont T Spits H. Stable long-term cultures of self-renewing B cells and their applications. Immunol Rev (2016) 270:65–77. 10.1111/imr.12395 26864105 PMC4755196

[28] Marks JD Hoogenboom HR Bonnert TP McCafferty J Griffiths AD Winter G. By-passing immunization. Human antibodies from V-gene libraries displayed on phage. J Mol Biol (1991) 222:581–97. 10.1016/0022-2836(91)90498-U 1748994

[29] Winter G Griffiths AD Hawkins RE Hoogenboom HR. Making antibodies by phage display technology. Annu Rev Immunol (1994) 12:433–55. 10.1146/annurev.iy.12.040194.002245 8011287

[30] Lowe D Jermutus L. Combinatorial protein biochemistry for therapeutics and proteomics. Curr Pharm Biotechnol (2004) 5:17–27. 10.2174/1389201043489585 14965207

[31] Peterson NC. Advances in monoclonal antibody technology: genetic engineering of mice, cells, and immunoglobulins. ILAR J (2005) 46:314–9. 10.1093/ilar.46.3.314 15953839

[32] Rader C Ritter G Nathan S Elia M Gout I Jungbluth AA The rabbit antibody repertoire as a novel source for the generation of therapeutic human antibodies. J Biol Chem (2000) 275:13668–76. 10.1074/jbc.275.18.13668 10788485

[33] Shim H. Therapeutic antibodies by phage display. Curr Pharm Des (2016) 22:6538–59. 10.2174/1381612822666160923113714 27669967

[34] Chen G Sidhu SS. Design and generation of synthetic antibody libraries for phage display. Methods Mol Biol (2014) 1131:113–31. 10.1007/978-1-62703-992-5_8 24515463

[35] Benhar I. Design of synthetic antibody libraries. Expert Opin Biol Ther (2007) 7:763–79. 10.1517/14712598.7.5.763 17477812

[36] Adams JJ Sidhu SS. Synthetic antibody technologies. Curr Opin Struct Biol (2014) 24:1–9. 10.1016/j.sbi.2013.11.003 24721448

[37] Knappik A Ge L Honegger A Pack P Fischer M Wellnhofer G Fully synthetic human combinatorial antibody libraries (HuCAL) based on modular consensus frameworks and CDRs randomized with trinucleotides. J Mol Biol (2000) 296:57–86. 10.1006/jmbi.1999.3444 10656818

[38] Rothe C Urlinger S Lohning C Prassler J Stark Y Jager U The human combinatorial antibody library HuCAL GOLD combines diversification of all six CDRs according to the natural immune system with a novel display method for efficient selection of high-affinity antibodies. J Mol Biol (2008) 376:1182–200. 10.1016/j.jmb.2007.12.018 18191144

[39] Tiller T Schuster I Deppe D Siegers K Strohner R Herrmann T A fully synthetic human Fab antibody library based on fixed VH/VL framework pairings with favorable biophysical properties. MAbs (2013) 5:445–70. 10.4161/mabs.24218 23571156 PMC4169037

[40] Shim H. Synthetic approach to the generation of antibody diversity. BMB Rep (2015) 48:489–94. 10.5483/BMBRep.2015.48.9.120 26129672 PMC4641231

[41] Flego M Ascione A Cianfriglia M Vella S. Clinical development of monoclonal antibody-based drugs in HIV and HCV diseases. BMC Med (2013) 11:4. 10.1186/1741-7015-11-4 23289632 PMC3565905

[42] Ozawa T Piao X Kobayashi E Zhou Y Sakurai H Andoh T A novel rabbit immunospot array assay on a chip allows for the rapid generation of rabbit monoclonal antibodies with high affinity. PLoS One (2012) 7:e52383. 10.1371/journal.pone.0052383 23300658 PMC3530603

[43] Starkie DO Compson JE Rapecki S Lightwood DJ. Generation of recombinant monoclonal antibodies from immunised mice and rabbits via flow cytometry and sorting of antigen-specific IgG+ memory B cells. PLoS One (2016) 11:e0152282. 10.1371/journal.pone.0152282 27022949 PMC4811437

[44] Bandeira N Pham V Pevzner P Arnott D Lill JR Automated de novo protein sequencing of monoclonal antibodies. Nat Biotechnol (2008) 26:1336–8. 10.1038/nbt1208-1336 19060866 PMC2891972

[45] Wine Y Boutz DR Lavinder JJ Miklos AE Hughes RA Hoi KH Molecular deconvolution of the monoclonal antibodies that comprise the polyclonal serum response. Proc Natl Acad Sci U S A (2013) 110:2993–8. 10.1073/pnas.1213737110 23382245 PMC3581903

[46] Shawler DL Bartholomew RM Smith LM Dillman RO. Human immune response to multiple injections of murine monoclonal IgG. J Immunol (1985) 135:1530–5. 3874237

[47] Schroff RW Foon KA Beatty SM Oldham RK Morgan AC Jr. Human anti-murine immunoglobulin responses in patients receiving monoclonal antibody therapy. Cancer Res (1985) 45:879–85. 3871353

[48] Huang Z Zhu W Meng Y Xia H. Development of new rabbit monoclonal antibody to progesterone receptor (Clone SP2): no heat pretreatment but effective for paraffin section immunohistochemistry. Appl Immunohistochem Mol Morphol (2006) 14:229–33. 10.1097/01.pai.0000157906.38495.31 16785796

[49] Tao GZ Nakamichi I Ku NO Wang J Frolkis M Gong X Bispecific and human disease-related anti-keratin rabbit monoclonal antibodies. Exp Cell Res (2006) 312:411–22. 10.1016/j.yexcr.2005.11.010 16343483

[50] Bystryn JC Jacobsen JS Liu P Heaney-Kieras J. Comparison of cell-surface human melanoma-associated antigens identified by rabbit and murine antibodies. Hybridoma (1982) 1:465–72. 10.1089/hyb.1.1982.1.465 6208141

[51] Feng L Wang X Jin H. Rabbit monoclonal antibody: potential application in cancer therapy. Am J Transl Res (2011) 3:269–74. 21633632 PMC3102571

